# Factors affecting critical care admission to a UK university hospital

**DOI:** 10.1186/cc11115

**Published:** 2012-03-20

**Authors:** A Tridente, A Chick, S Keep, S Furmanova, S Webber, DC Bryden

**Affiliations:** 1 Sheffield Teaching Hospitals, Sheffield, UK; 2 University Hospital Wales, Cardiff, UK

## Introduction

Access to critical care is limited, with disparity existing between availability and demand. Guidance to inform triage decisions has been published but may no longer reflect current pressures [1,2]. We aimed to identify a set of criteria able to reliably predict likelihood of admission to a critical care unit in a large UK tertiary care centre.

## Methods

Consecutive patient referrals were prospectively enrolled in a review cohort. Data were collected using a predefined case report form (CRF). The CRF included information on the referral, acute physiological parameters, hospital length of stay (LOS), demographic and functional status, dependency and comorbidities. Logistic regression was performed to identify factors predicting admission, employing STATA [3].

## Results

Between 17 July and 27 November 2011, 201 patients were referred to critical care, of whom 85 (42.7%) were declined. Median age (interquartile range) was 67 (54 to 79) years, 121 (60.8%) were male, median LOS (interquartile range) was 1 (1 to 3) day. Age, gender, ethnic origin, LOS, referral reason, and markers of acute physiological derangement did not impact on likelihood of admission to critical care. Odds ratios (95% CIs) for admission were 3.1 (1.72 to 5.56) for exercise tolerance >100 yards (*P *< 0.001), 3.03 (1.56 to 5.89) for self-caring status (*P *= 0.001), 0.38 (0.2 to 0.71) for house-bound status (*P *= 0.003), 0.28 (0.1 to 0.76) for wheelchair-bound status (*P *= 0.013), 0.41 (0.23 to 0.74) for cardiovascular (*P *= 0.003), 0.36 (0.18 to 0.72) for renal system (*P *= 0.004), 0.34 (0.14 to 0.85) for malignant (*P *= 0.021), and 0.49 (0.25 to 0.94) for neurological (*P *= 0.033) comorbidities, respectively.

## Conclusion

Our data suggest that critical care admission decisions are made based mainly on the assessment of patients' pre-morbid state and functional capacity, rather than on the extent of acute physiological derangement. This behaviour is more consistent with the application of a prioritization model, defining those patients who will benefit most from critical care admission (Priority 1) to those who will not benefit at all (Priority 4) and consistent with pressured resources, rather than an objective parameters model or a diagnostic model [1].
